# Biomarkers of evasive resistance predict disease progression in cancer patients treated with antiangiogenic therapies

**DOI:** 10.18632/oncotarget.7915

**Published:** 2016-03-04

**Authors:** Andreas Pircher, Karin Jöhrer, Florian Kocher, Normann Steiner, Ivo Graziadei, Isabel Heidegger, Renate Pichler, Nicolai Leonhartsberger, Christian Kremser, Johann Kern, Gerold Untergasser, Eberhard Gunsilius, Wolfgang Hilbe

**Affiliations:** ^1^ Department of Internal Medicine V, Hematology and Oncology, Medical University Innsbruck, Innsbruck, Austria; ^2^ Tyrolean Cancer Research Institute, Innsbruck, Austria; ^3^ Department of Internal Medicine II, Gastroenterology and Hepatology, Medical University Innsbruck, Innsbruck, Austria; ^4^ Department of Urology, Medical University Innsbruck, Innsbruck, Austria; ^5^ Department of Radiology, Medical University Innsbruck, Innsbruck, Austria; ^6^ Department of Oncology, Hematology and Palliative Care Wilhelminenspital, Vienna, Austria

**Keywords:** angiogenesis, antiangiogenic therapies, placental growth factor (PlGF), vascular endothelial growth factor (VEGF), Robo4

## Abstract

Numerous antiangiogenic agents are approved for the treatment of oncological diseases. However, almost all patients develop evasive resistance mechanisms against antiangiogenic therapies. Currently no predictive biomarker for therapy resistance or response has been established. Therefore, the aim of our study was to identify biomarkers predicting the development of therapy resistance in patients with hepatocellular cancer (*n* = 11), renal cell cancer (*n* = 7) and non-small cell lung cancer (*n* = 2). Thereby we measured levels of angiogenic growth factors, tumor perfusion, circulating endothelial cells (CEC), circulating endothelial progenitor cells (CEP) and tumor endothelial markers (TEM) in patients during the course of therapy with antiangiogenic agents, and correlated them with the time to antiangiogenic progression (aTTP). Importantly, at disease progression, we observed an increase of proangiogenic factors, upregulation of CEC/CEP levels and downregulation of TEMs, such as Robo4 and endothelial cell-specific chemotaxis regulator (ECSCR), reflecting the formation of torturous tumor vessels. Increased TEM expression levels tended to correlate with prolonged aTTP (ECSCR high = 275 days vs. ECSCR low = 92.5 days; *p* = 0.07 and for Robo4 high = 387 days vs. Robo4 low = 90.0 days; *p* = 0.08). This indicates that loss of vascular stabilization factors aggravates the development of antiangiogenic resistance. Thus, our observations confirm that CEP/CEC populations, proangiogenic cytokines and TEMs contribute to evasive resistance in antiangiogenic treated patients. Higher TEM expression during disease progression may have clinical and pathophysiological implications, however, validation of our results is warranted for further biomarker development.

## INTRODUCTION

Angiogenesis, the formation of blood vessels, is a hallmark of cancer that significantly contributes to cancer progression and metastasis [1]. Increased tumor angiogenesis consequently limits prognosis and overall survival of cancer patients [1]. However, the success of blocking angiogenesis by inhibiting vascular endothelial growth factor (VEGF) is limited by insufficient efficacy and the development of resistance [2–5]. Preclinical studies suggest multiple markers and mechanisms potentially involved in intrinsic or acquired resistance against antiangiogenic therapies (reviewed in [5, 6]), but only few clinical studies evaluate surrogate markers during therapy.

An important driver for development of therapy resistance is the increase of tumor hypoxia during antiangiogenic treatment [7] leading to the upregulation of the transcription factor hypoxia-inducible factor 1 alpha (HIF-1A). HIF-1A activates survival pathways in tumor cells and increases production of angiogenic growth factors such as VEGF or fibroblast growth factor (FGF) and others, thereby inducing more aggressive tumor growth, influencing endothelial cell behavior, and promoting therapy resistance [7, 8]. For example, when VEGF is neutralized by antibodies (e.g. Bevacizumab) or VEGF-receptor (VEGFR) signaling is inhibited by receptor-tyrosine kinase inhibitors (e.g. Sunitinib, Sorafenib), compensatory angiogenic pathways and cytokines can be upregulated thereby stimulating resident endothelial cells [5].

Furthermore, circulating endothelial progenitor cells (CEPs) can be recruited from the bone marrow and contribute to new blood vessel formation in the tumor (vasculogenesis) [9, 10]. Additionally, tumors treated with antiangiogenic therapy show a tendency towards coopting already existing healthy blood vessels and thereby securing nutrient and oxygen supply independently of tumor angiogenesis [5, 11]. Moreover, morphological analyses of tumor vessels revealed changes in pericyte coverage and vessel structure, which may render the vessels resistant to antiangiogenic therapies and influence blood perfusion and pressure inside the tumor [5]. In the clinical setting, changes of circulating angiogenic growth factors correlate with patient response and benefit from antiangiogenic therapy [3, 12]. However, limited information on the development of other resistance mechanisms, such as upregulation of alternative angiogenic growth factors, is available. Furthermore additional studies are urgently needed to achieve a more comprehensive view on therapy resistance in patients.

**Figure 1:**
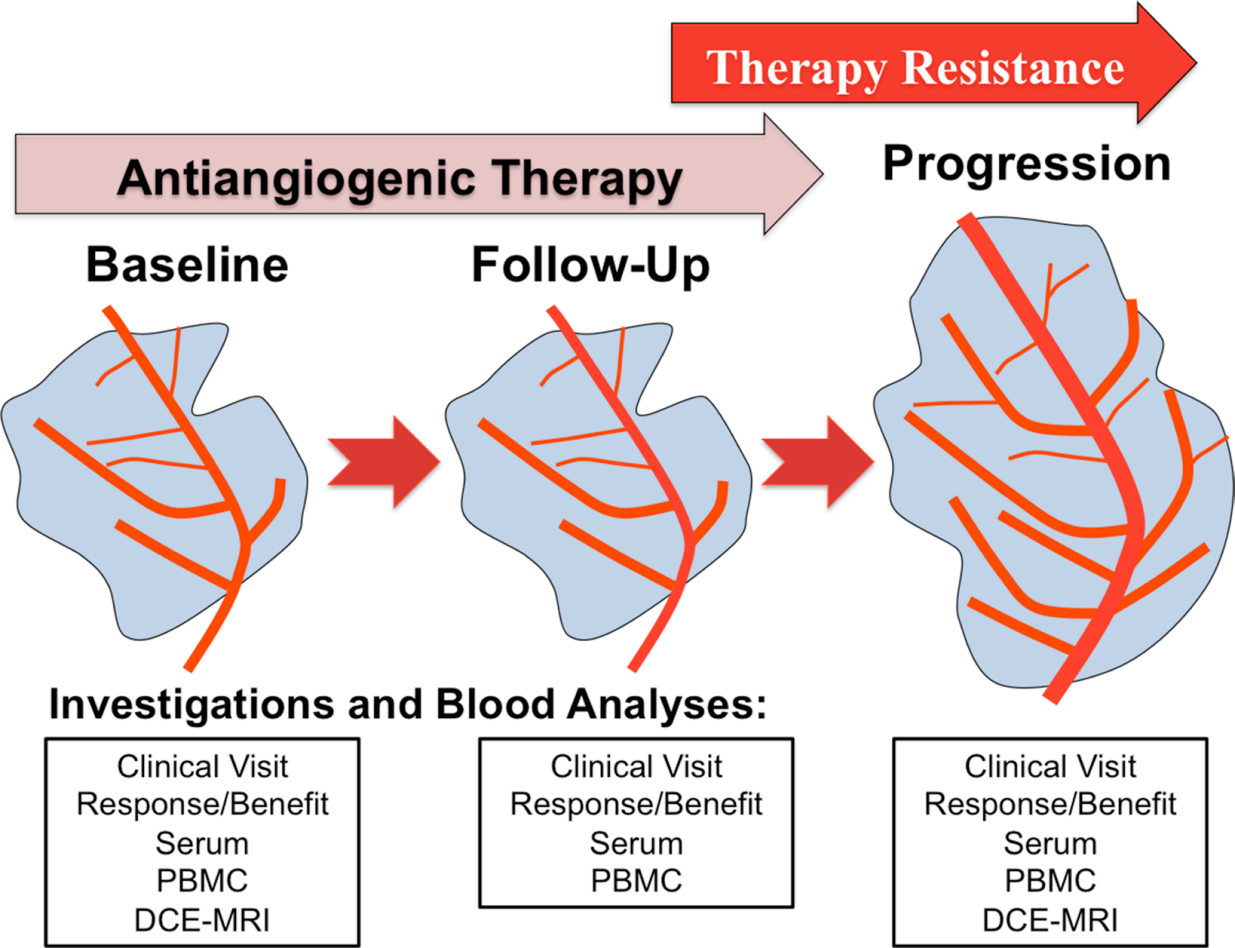
Study synopsis showing the planned investigations at each clinical visit (baseline, follow-up and disease progression) *Abbreviations:* peripheral blood mononuclear cells (PBMC), dynamic contrast enhanced magnetic response imaging (DCE-MRI).

In addition, identifying those patients who might benefit from antiangiogenic therapies is of utmost clinical importance. As a consequence, predictive biomarkers are indispensable tools to choose the most effective drugs and protect patients from unnecessary side effects. The aim of the present study was to generate a profile of possible mechanisms of resistance in patients with renal cell cancer (RCC), hepatocellular cancer (HCC) and non-small cell lung cancer (NSCLC) treated with sunitinib, sorafenib or bevacizumab thereby comparing and correlating changes in angiogenic growth factors, circulating cell populations and tumor endothelial markers (TEM) to disease progression (Study plan shown in Figure 1).

## RESULTS

### Patients' characteristics

The median age of the study population was 66 years (range 34–81 years), and the male/female distribution was 17/3. Serum samples, peripheral blood mononuclear cells (PBMC) and circulating cells were available from all 20 patients. Regarding progression free survival (PFS) and time to antiangiogenic progression (aTTP) 19 patients were evaluable. Detailed patients' characteristics are shown in Table 1.

**Table 1:** Patient demographics and baseline characteristics (*n* = 20)

Characteristics	*N*	%
Male	17	85
Median age (range), years	66 (34–81)	
Tumor type		
Non-small cell lung cancer (NSCLC)	2	10
Hepatocellular carcinoma (HCC)	11	55
Renal cell cancer (RCC)	7	35
Performance status (WHO grade)		
0 – 1	20	100
Treatment		
Bevacizumab	2	10
Sorafenib	12	60
Sunitinib	6	30
NSCLC (*n* = 2)		
Histology		
Adenocarcinoma	2	100
First line therapy		
Cisplatin/Gemcitabine/Bevacizumab	1	50
Cisplatin/Docetaxel/Bevacizumab	1	50
RCC (*n* = 7)		
Previous therapies*		
Tumornephrectomy	7	100
Metastasectomy	5	71
Radiation of metastasis	4	57
Systemic palliative therapy		
First line	5	71
Second line	2	29
HCC (*n* = 11)		
Previous therapies		
Locoablative therapy*	7	64
Surgery	5	
Chemoembolisation	7	
Radiofrequency ablation (RFA)	5	
No locoablative therapy	4	36
Underlying liver disease		
Hepatitis B	3	27
Hepatitis C	1	9
(non) alcoholic fatty liver disease	5	46
Kryptogenic	2	18

* The majority of HCC and RCC received different locoablative therapies prior to the start of antiangiogenic therapy.

All patients showed at least short-term response upon antiangiogenic therapy. In the HCC cohort (*n* = 11) six patients showed stable disease (SD), two patients partial response (PR) and two patients showed progressive disease shortly after study inclusion. Of note, these two patients underwent sorafenib therapy for at least two months before study inclusion. In the RCC group (*n* = 7), two patients showed SD, four patients showed PR, and one patient showed progressive disease two months after study inclusion. The included NSCLC patients (*n* = 2) received bevacizumab maintenance therapy and showed SD and PR, respectively.

### Circulating levels of angiogenesis-related molecules change from baseline to disease progression

**Figure 2:**
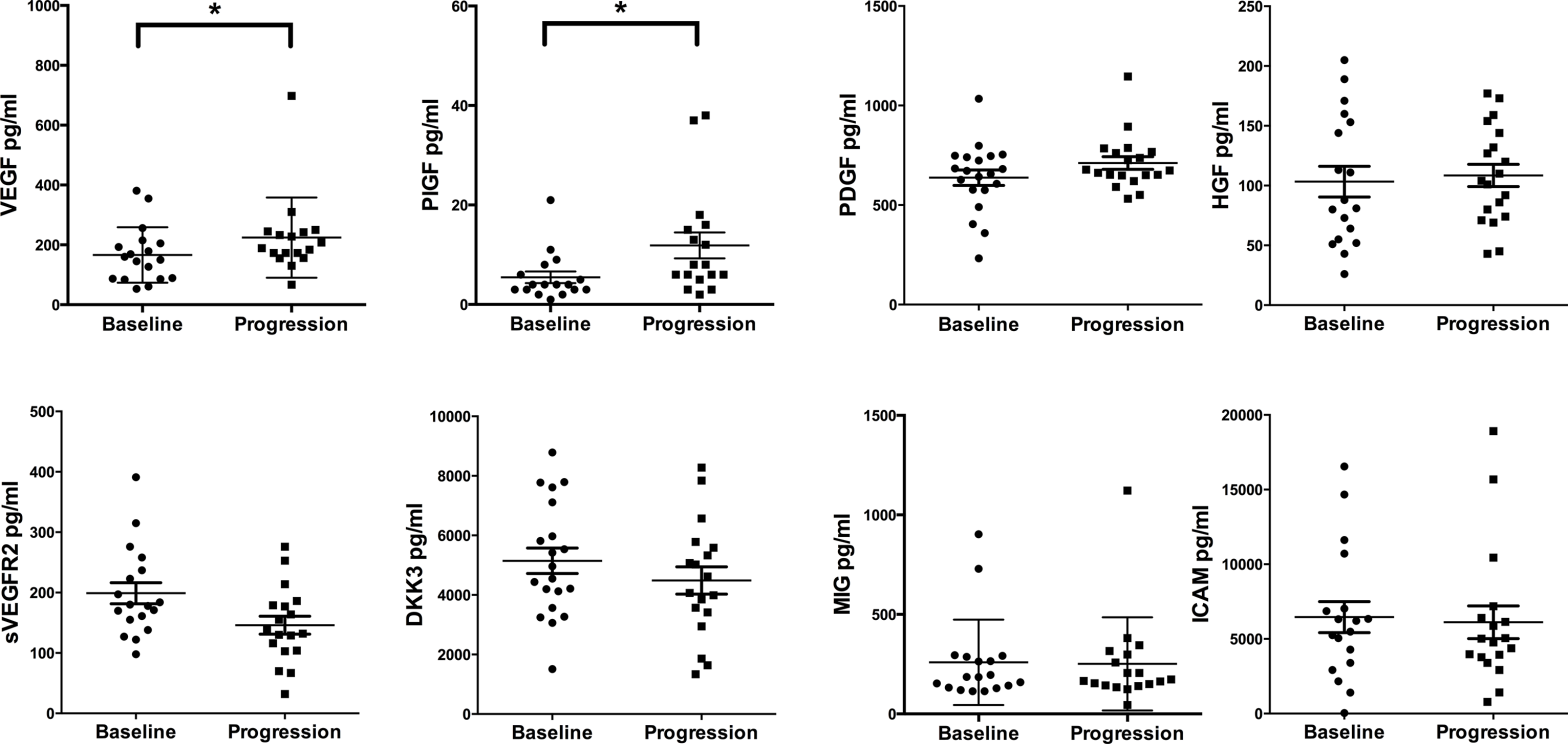
Cytokine analyses comparing baseline investigations with disease progression Paired serum samples were analyzed from patients at baseline and disease progression. X-axis depicts the time points of measurements baseline versus disease progression. Y-axis depicts the measured cytokine in picograms per milliliter (pg/ml). *p ≤ 0.05. *Abbreviations:* vascular endothelial growth factor-A (VEGF), placental growth factor (PlGF), platelet derived growth factor (PDGF), hepatocyte growth factor (HGF), soluble vascular endothelial growth factor receptor2 (sVEGFR2), dickkopf3 (DKK3), monokine induced by gamma interferon (MIG), intracellular adhesion molecule1 (ICAM).

We measured serum levels of proangiogenic cytokines at baseline and disease progression and found that VEGF, PDGF, PlGF and HGF increased during the course of therapy (Figure 2). In contrast, we observed that cytokines like sVEGFR2, DKK3, MIG and ICAM decreased during the course of therapy (Figure 2).

Of note, the serum concentration of VEGF was significantly increased at disease progression (from median 155 pg/mL [range 53–381 pg/ml, *n* = 18] baseline to 189 pg/ml [range 67–698 pg/ml, *n* = 17] at disease progression, *p* = 0.04), whereas sVEGFR2 showed a moderate decrease (from 179 pg/ml [range 98–391 pg/mL, *n* = 18] at baseline to 135 pg/ml [range 32–267 pg/mL, *n* = 18] at disease progression, *p* = 0.08). A significant growth factor increase was observed for PlGF (from median 4 pg/mL [range 1–21 pg/ml, *n* = 17] at baseline to 8 pg/ml [range 2–38 pg/ml, *n* = 17] at disease progression, *p* = 0.05). PDGF tended to be increased (from median 656 pg/mL [range 232–1034 pg/ml, *n* = 19] at baseline to 690 pg/ml [range 532–1146 pg/ml, *n* = 19] at disease progression, *p* = 0.06), and DKK3 tended to be decreased (from median 4754 pg/mL [range 1510–8758 pg/ml, *n* = 18] at baseline to 4347 pg/ml [range 1335–8278 pg/ml, *n* = 18] at disease progression, *p* = 0.08), although those changes did not reach significance. For HGF, ICAM, IL-10, IL-12p40, IL-12p70, IP-10, FGF and MIG no significant changes were detected (Figure 2 and Supplementary Figure 1).

### Levels of circulating endothelial cells (CEC) and progenitor cells (CEP) increase from baseline to disease progression

**Figure 3:**
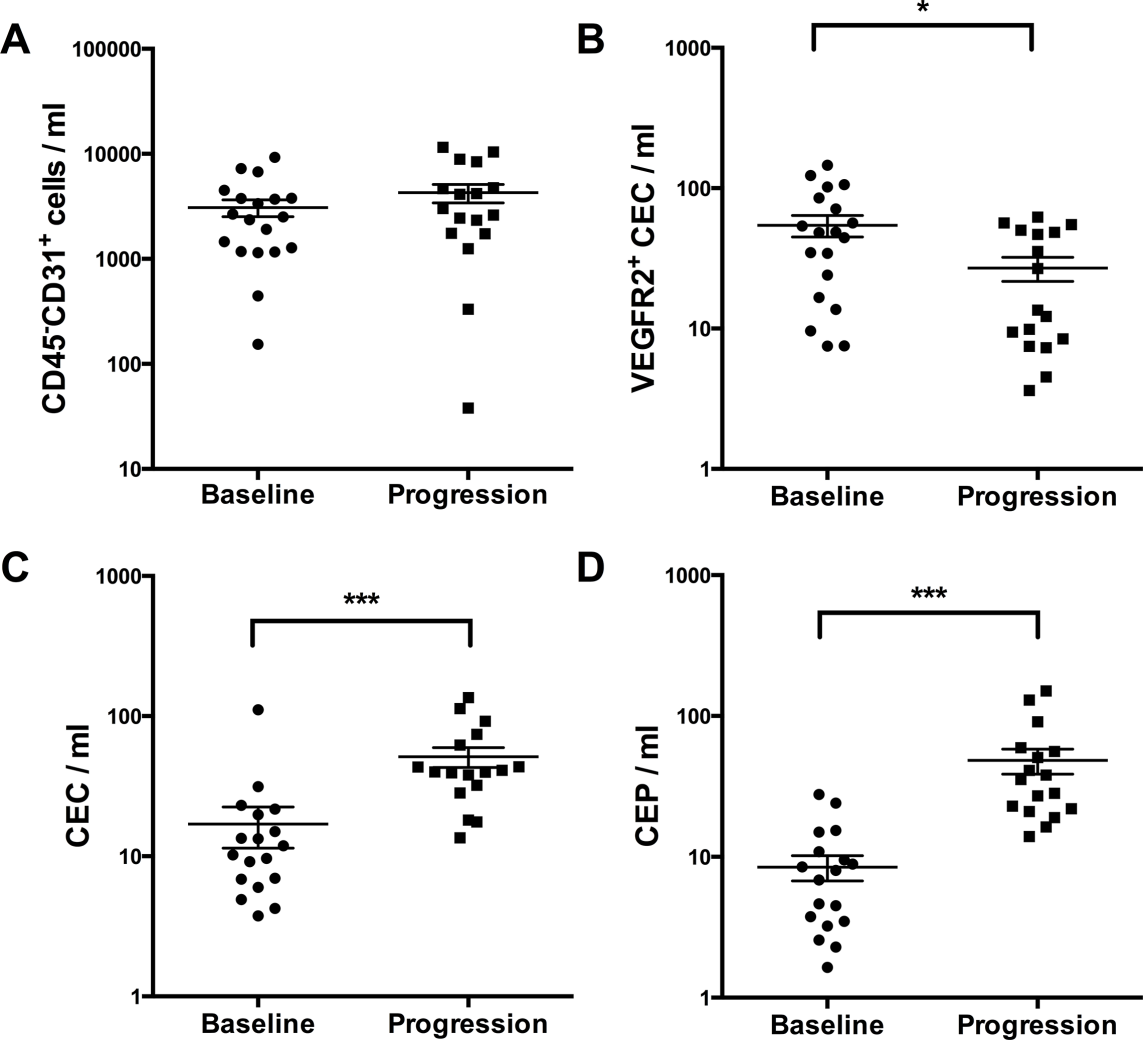
Measurement of different circulating cell populations at baseline and disease progression (A–D) by multicolor FACS analysis X-axis depicts the time points of measurements at baseline versus disease progression. Y-axis depicts the measured cell populations cells/microliter. *p ≤ 0.05; ***p ≤ 0.001. *Abbreviations:* circulating endothelial cells (CEC), circulating endothelial progenitor cells (CEP), vascular endothelial growth factor receptor2 (VEGFR2).

Levels of CECs and CEPs are indicative of high vascular turnover and potential candidates for monitoring antiangiogenic therapies [13]. As shown in Figure 3, CEC and CEP changed significantly during therapy. In detail CEC levels increased markedly at disease progression (from median 11.88 cells/mL [range 0–111.2 cells/ml, *n* = 17] at baseline to 40.00 cells/ml [range 13.56–135.4 cells/ml, *n* = 17] at disease progression, *p* < 0.001), whereas the CD45^−^CD31^+^ cell populations remained relatively stable (from median 2360 cells/mL [range 154–9239 cells/ml, *n* = 17] at baseline to 3021 cells/ml [range 38–11535 cells/mL, *n* = 17] at disease progression, *p* = 0.07). CEP levels increased during the course of therapy (from median 4.63 cells/mL [range 0–27.68 cells/ml, *n* = 17] at baseline to 35.44 cells/ml [range 13.94–150.9 cells/ml, *n* = 17] at disease progression, *p* < 0.001). In contrast, VEGFR2^+^CEC numbers were significantly lower at disease progression (from median 48.28 cells/mL [range 7.50–145.8 cells/mL, *n* = 17] baseline to 13.54 cells/ml [range 3.62–62.28 cells/ml, *n* = 17] at disease progression, *p* = 0.003).

### TEMs (ECSCR, Robo4, Clec14) expression levels decrease from baseline to disease progression

**Figure 4:**
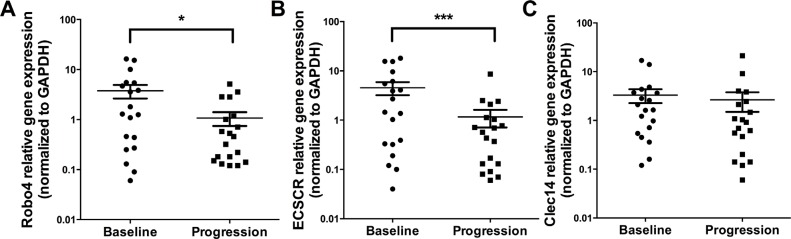
Expression levels of TEMs at baseline and disease progression by qPCR. Analyses are shown for Robo4 (A), ECSCR (B) and Clec14 (C) by qPCR X-axis depicts the time points of measurements at baseline versus disease progression. Y-axis depicts relative gene expression of Robo4, ECSCR and Clec14 normalized to GAPDH. *p ≤ 0.05; ***p ≤ 0.001. *Abbreviations:* endothelial cell-specific chemotaxis regulator (ECSCR), tumor endothelial marker (TEM), glyceraldehyde 3-phosphate dehydrogenase (GAPDH).

TEM have been shown to be selectively expressed by tumor endothelial cells [14] and to have important biological functions (e.g. Robo4 inhibits VEGFR2 signaling) [15]. Therefore, we analyzed Robo4, Clec14 and ECSCR expression levels in PBMC, which decreased significantly at disease progression (Figure 4): Robo4 expression decreased significantly from baseline to disease progression (median expression at baseline 1.29 [range 0.06–16.28], *n* = 19, to 0.46 [range 0.12–5.12], at progression, *n* = 19, *p* = 0.04). Also ECSCR levels dropped from baseline to progression (baseline median expression was 1.45 [range 0.04–18.02], *n* = 19) compared to progression (median 0.57 [range 0.06–8.67], *n* = 19, *p* = 0.009). Similarly, higher expression levels of Clec14 were observed at baseline (median 1.64 [range 0.12–17.01], *n* = 19), compared to disease progression (median 0.91 [range 0.06 – 21.16], *n* = 19, *p* = 0.09).

### Changes of DCE-MRI perfusion from baseline to disease progression

**Figure 5:**
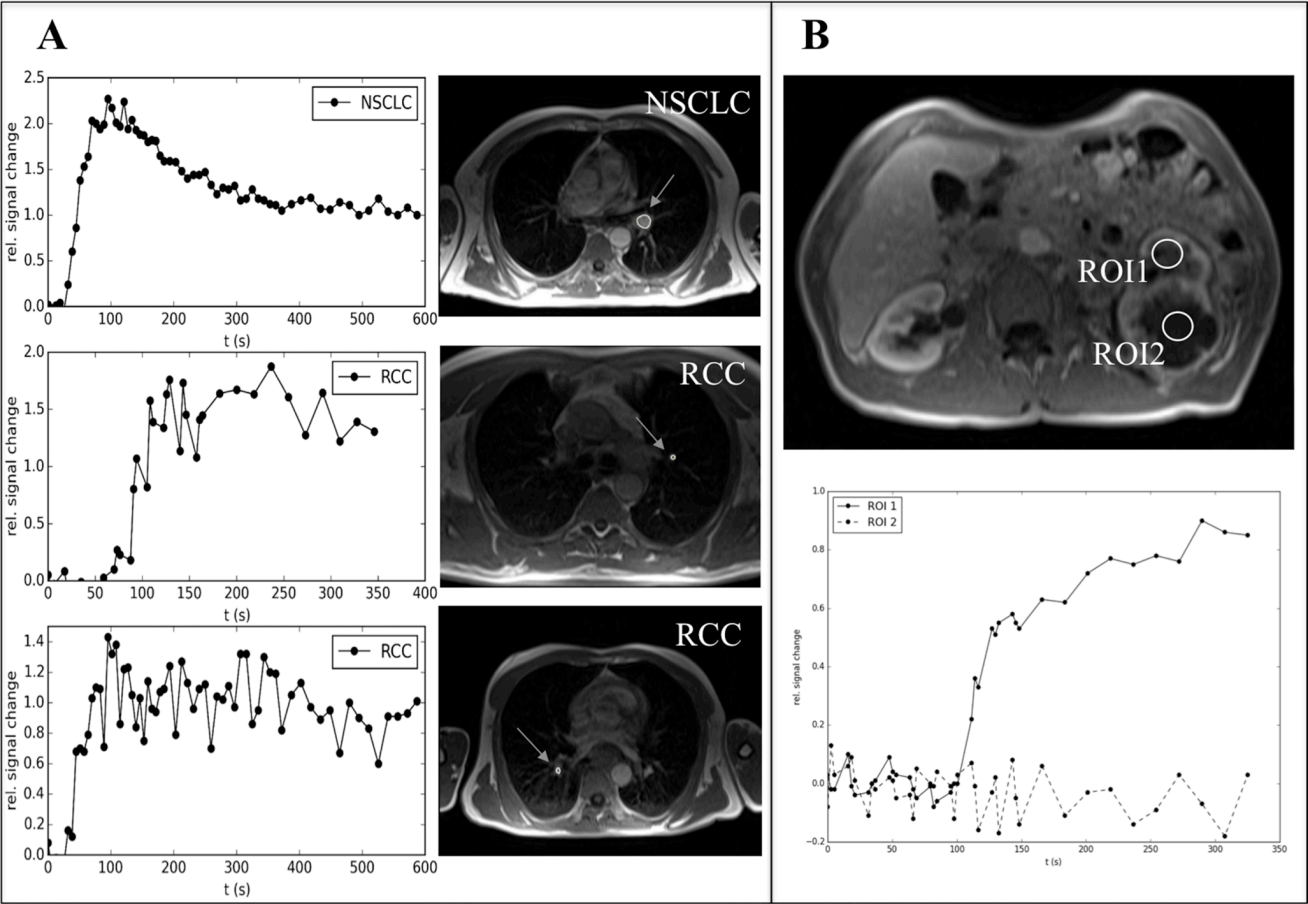
Representative examples of DCE-MRI of patients benefitting from antiangiogenic therapies (baseline assessment) (**A**) On the left side contrast media uptake curves and on the right side corresponding MRI images of the tumor at baseline are shown (the circle and arrows indicate the tumor). (**B**) Two regions of interest (ROI1 and 2) of an extensive primary RCC during antiangiogenic therapy are shown (upper picture). ROI1 shows contrast media uptake (type3 curve, normalization during sunitinib therapy) while ROI2 reflects necrosis in the tumor with almost no perfusion/permeability (lower picture). *Abbreviations:* dynamic contrast enhanced magnetic resonance imaging (DCE-MRI), renal cell cancer (RCC), non-small cell lung cancer (NSCLC), region of interest (ROI).

**Figure 6:**
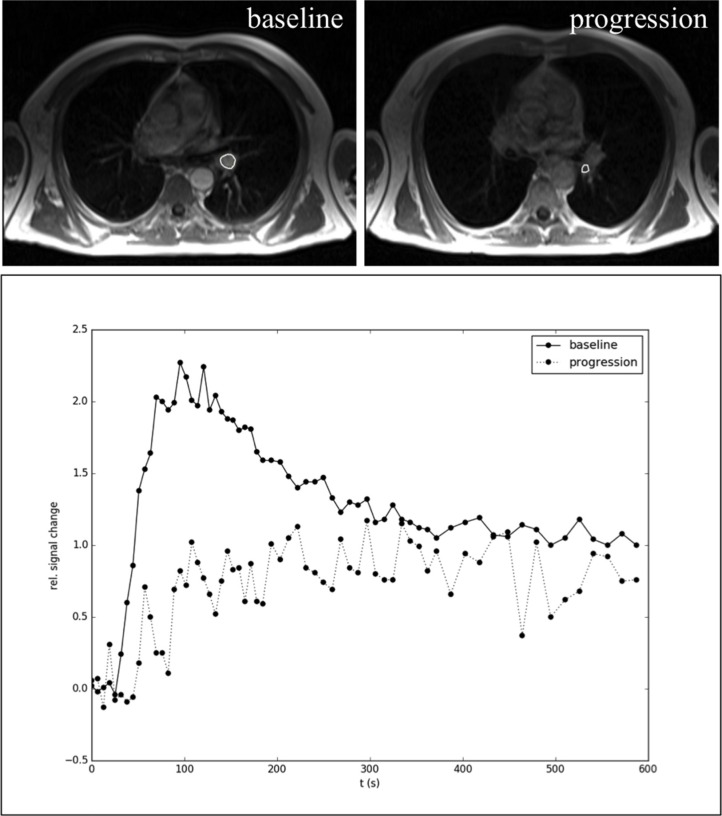
Representative example of DCE-MRI in a NSCLC patient during bevacizumab maintenance therapy at baseline and disease progression In the upper row representative MRI images of the tumor at baseline and progression are shown (the circles indicate the tumor). Below the corresponding curves and changes in contrast media uptake behavior are depicted. The upper curve at baseline represents a type 3 curve, which correlates with an increased tumor microcirculation (permeability/perfusion) induced by the antiangiogenic agent (vessel normalization). At disease progression the contrast uptake curve was changed to a type 1 curve, which corresponds to lower permeability/perfusion. *Abbreviations:* dynamic contrast enhanced magnetic resonance imaging (DCE-MRI), non-small cell lung cancer (NSCLC), region of interest (ROI).

We analyzed tumor blood perfusion by DCE-MRI to investigate the *in vivo* efficacy of antiangiogenic therapies [16]. Baseline DCE-MRI was performed in 10/20 patients. Our data show perfusion normalization during antiangiogenic therapy. Representative contrast agent enhancement curves at baseline are shown in Figure 5 reflecting more type 2 and type 3 curves (Figure 5A). Furthermore, we specifically depicted intra-tumor perfusion/permeability heterogeneity. The necrotic area (ROI2, Figure 5B) showed no contrast media uptake in comparison to the remaining active tumor tissue, where normalized contrast media uptake is depicted (ROI1, Figure 5B). The low recruitment number was due to the time-delayed coordination with the results obtained from the CT scans, where, as a result from the scans, most patients at progression were switched to another therapy or ongoing antiangiogenic therapy was stopped. Thus, DCE-MRI could not be performed in the preplanned timeframes. However, in the two available patients significant changes in tumor perfusion were observed (Figure 6).

### Correlation of the analyzed markers, techniques and time points

Correlation analyses revealed associations of different angiogenic cytokines including ICAM with sVEGFR2 (*r* = −0.60, *p* = 0.005, *q* = 0.05) as well as sVEGFR2 with DKK3 (*r* = 0.16, *p* = 0.015, *q* = 0.075). Indeed, significant correlations between circulating cell populations, angiogenic factors and TEMs were identified. For example baseline VEGF expression correlated with CEP levels (*r* = 0.73, *p* = 0.003, *q* = 0.03) reflecting that VEGF is an important CEP attracting factor [17]. Interestingly, at disease progression CEP levels correlated with PlGF change from baseline to progression (*r* = 0.577, *p* = 0.015, *q* = 0.441). After correction for multiple testing, by using the false discovery rate (FDR) [18] some previous significant correlations failed to reach the pre-specified level of significance. However, it can be argued that such marker changes are still of biological interest. Furthermore, correlation analyses of marker alterations (defined as: progression level subtracted baseline level) with other analyses are summarized in Table 2.

**Table 2:** Correlation analysis of changes of measured parameters from baseline to disease progression during antiangiogenic therapy (correlation analysis of level changes of cytokines, circulation cell populations and TEMs)

	VEGF	HGF	DKK3	PlGF	Robo4	ECSCR	Clec14
**CEC**							
Pearson *r* coefficient	0.052	**−0.632**	**−0.422**	−0.221	0.045	**−0.045**	−0.143
*p* value	0.848	**0.010**	**0.072**	0.428	0.859	**0.038**	0.572
FDR; *q* value	0.859	**0.090**	**0.216**	0.859	0.859	**0.171**	0.859
**CEP**							
Pearson *r* coefficient	0.202	−0.007	−0.040	**0.501**	−0.121	−0.211	−0,240
*p* value	0.454	0.098	0.872	**0.049**	0.632	0.402	0.338
FDR; *q* value	0.817	0.441	0.981	**0.441**	0.948	0.817	0.817
**sVEGFR**							
Pearson *r* coefficient	**0.624**	0.123	0.102	−0.227	−0.171	−0.079	−0.260
*p* value	**0.006**	0.626	0.661	0.380	0.472	0.741	0.268
FDR; *q* value	**0.054**	0.950	0.950	0.950	0.950	0.950	0.950
**Robo4**							
Pearson *r* coefficient	0.018	0.034	0.217	0.288	/	**0.681**	**0.544**
*p* value	0.943	0.895	0.358	0.263	/	**0.001**	**0.013**
FDR; *q* value	0.944	0.944	0.646	0.646	/	**0.008**	**0.052**
**ECSCR**							
Pearson *r* coefficient	−0.104	0.089	**0.520**	0.199	**0.681**	/	**0.445**
*p* value	0.476	0.787	**0.019**	0.444	**0.001**	/	**0.049**
FDR; *q* value	0.635	0.787	**0.076**	0.635	**0.008**	**/**	**0.130**
**PFS**							
Pearson *r* coefficient	0.062	−0.308	−0.283	0.226	0.203	0.224	0.275
*p* value	0.813	0.556	0.634	0.882	0.944	0.854	0.793
FDR; *q* value	0.986	0.986	0.986	0.986	0.986	0.986	0.986
**aTTP**							
Pearson *r* coefficient	0.001	−0.154	−0.117	0.061	0.017	0.048	0.065
*P* value	0.998	0.556	0.634	0.822	0.944	0.845	0.793
FDR; *q* value	0.998	0.998	0.998	0.998	0.998	0.998	0.998

Abbreviations: vascular endothelial growth factor-A (VEGF), hepatocyte growth factor (HGF), dickkopf3 (DKK3), placental growth factor (PlGF), endothelial cell-specific chemotaxis regulator (ECSCR), circulating endothelial cells (CEC), circulating endothelial progenitor cells (CEP), soluble vascular endothelial growth factor receptor2 (sVEGFR2), time to antiangiogenic progression (aTTP), progression free survival (PFS), tumor endothelial marker (TEM), false discovery rate (FDR).

### Correlation and regression analyses of the potential biomarkers with aPTT and PFS

**Figure 7:**
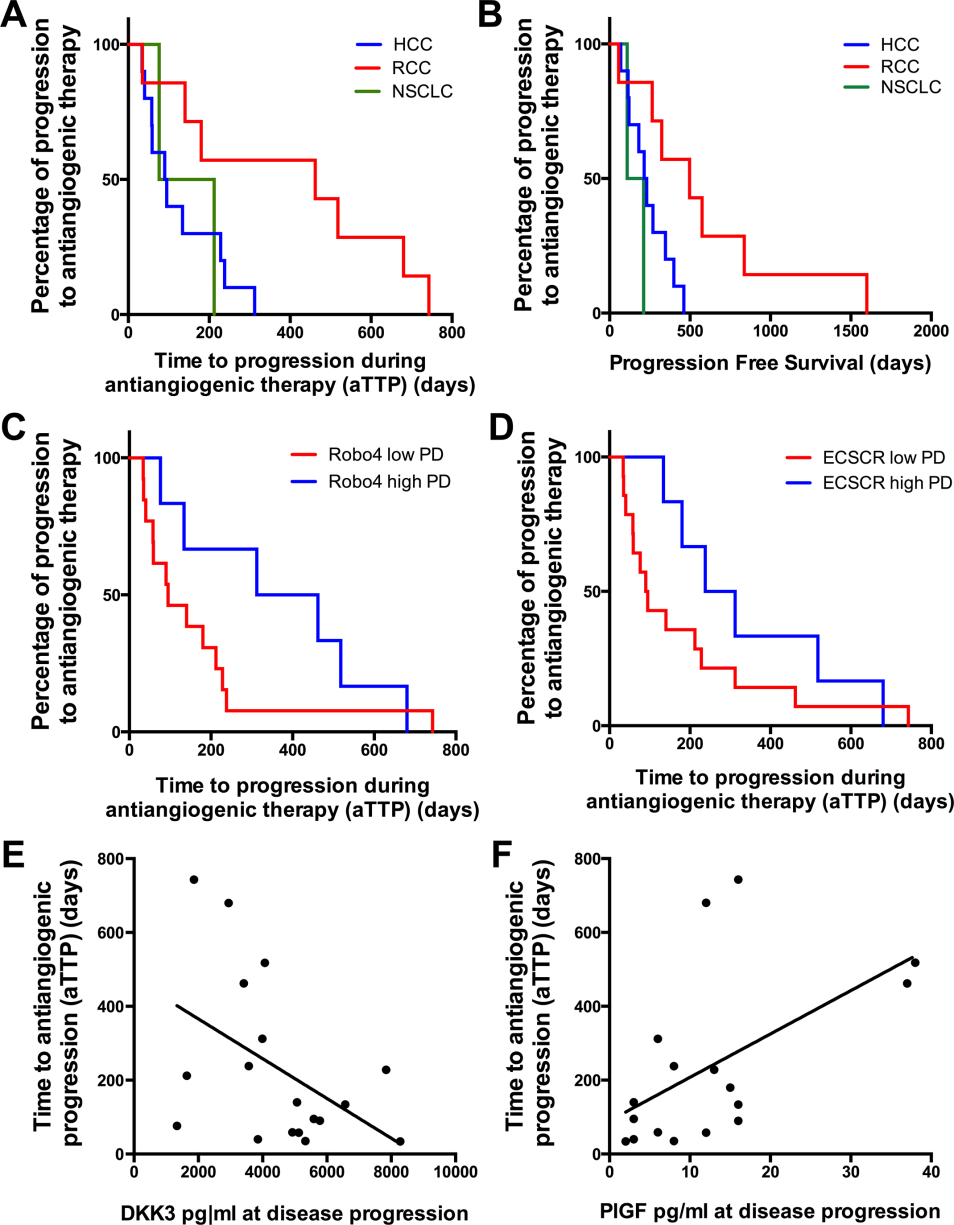
Kaplan-Meier curves showing the time to aTTP and PFS (**A**) and (**B**) show aTTP and PFS according to each tumor type. (A) aTTP (128 days [range 34–312 days] in HCC patients [*n* = 10], 394 days [range 35–743 days] in RCC patients [*n* = 7] and 144 days [range 76–212 days] in NSCLC patients [*n* = 2]); (B) PFS (241 days [range 69–462 days] in HCC patients [*n* = 10], 493 days [range 56–1599 days] in RCC patients [*n* = 7] and 161 days [range 109–212 days] for the NSCLC patients [*n* = 2]). (**C**) and (**D**) show the correlation of a high Robo4 (C) and ECSCR (D) expression at disease progression (PD) with a prolonged aPTT (Robo4 high = 387 days aPTT vs Robo4 low = 90.0 days, *p* = 0.08; ECSCR high = 275 days aPTT vs ECSCR low = 92.5 days, *p* = 0.07). (**E**) and (**F**) depict linear regression analysis showing a significant correlation of low DKK3 (E) and high PlGF (F) levels at disease progression with a prolonged aPTT (DKK3 levels at disease progression: *r*^2^ = 0.21, *p* = 0.05; PlGF levels at disease progression: *r*^2^ = 0.30, *p* = 0.02). (A–C): X-axis depicts the time of PFS and aPTT (days). (D–E): X-axis depicts the cytokine levels of DKK3 and PlGF at disease progression. (A–F): Y-axis depicts the percentage of patients showing a disease progression. *p ≤ 0.05; ***p ≤ 0.001. *Abbreviations:* time to antiangiogenic progression (aTTP), progression free survival (PFS), dickkopf3 (DKK3), placental growth factor (PlGF), endothelial cell-specific chemotaxis regulator (ECSCR), hepatocellular cancer (HCC), renal cell cancer (RCC), non-small cell lung cancer (NSCLC), progressive disease (PD).

With respect to clinical outcome, the median time to antiangiogenic progression (aTTP) was 128 days (range 34–312 days) in HCC patients, 394 days (range 35–743 days) in RCC patients and 144 days (range 76–212 days) in NSCLC patients, respectively (Figure 7A). The median PFS for the HCC cohort was 241 days (range 69–462 days), 493 days (range 56–1599 days) for the RCC patients and for the NSCLC patients during bevacizumab maintenance therapy 161 days (range 109–212 days) (Figure 7B). The PFS data are in line with the approval studies of the used agents [19−21].

Regression and correlation analyses of the analyzed markers with the aPTT and PFS revealed that neither marker alterations nor baseline levels correlated with the clinical end-points. However, correlations of marker expression levels at disease progression correlated with aPTT. We found that higher Robo4 and ECSCR expression levels at disease progression tended to be associated with a prolonged aPTT (Robo4 high = 387 days aPTT versus Robo4 low = 90.0 days, p = 0.08 [Figure 7C] and ECSCR high = 275 days aPTT versus ECSCR low = 92.5 days, *p* = 0.07 [Figure 7D]). However, the difference did not reach statistical significance. Furthermore cytokine levels like higher PlGF expression and a lower DKK3 expression at disease progression were associated with prolonged aPTT in a linear regression model (DKK3 levels at disease progression: *r*^2^ = 0.21, *p* = 0.05 [Figure 7E], PlGF levels at disease progression: *r*^2^ = 0.30, *p* = 0.02 [Figure 7F]).

## DISCUSSION

Several modes of antiangiogenic resistance have been postulated and studied in preclinical models [5, 22]. However, in the clinical setting only a few studies examined a comprehensive panel of possible resistance mechanisms throughout an ongoing antiangiogenic treatment [12, 23]. Hence, the presented study includes one of the broadest scientific programs accompanying the routine use of antiangiogenic therapies based on blood sample collection and functional imaging by DCE-MRI.

Intensive proangiogenic cytokine measurements for biomarker development during antiangiogenic therapies, either with monoclonal antibodies or tyrosine kinase inhibitors (TKI) targeting VEGF/R signaling, have been performed previously [12, 23−28]. Most of these studies reported an upregulation of proangiogenic factors like VEGF, PDGF, PlGF and HGF during therapy [23, 24, 29], which may reflect an increase of tumor hypoxia during antiangiogenic therapy. Our findings confirm upregulation of proangiogenic cytokines during antiangiogenic treatment. Tumor hypoxia is followed by compensation and restoration mechanisms to regain blood and nutrient supply via upregulation of other proangiogenic factors [5, 12]. Therefore, the upregulation of alternative proangiogenic signaling pathways similar in function of VEGF/R signaling is a potential mechanism of resistance e.g. PlGF was significantly upregulated during ramucirumab (VEGFR2 monoclonal antibody) therapy in HCC patients [30]. Indeed, targeting upregulated cytokines like PlGF, FGF or PDGF as well as the use of inhibitors targeting multiple pathways are currently under preclinical and clinical investigation for treatment of tumors resistant to VEGF-targeted therapy [31−33]. We detected an inverse correlation of VEGF and sVEGFR2, which has been described as a typical effect reflecting the efficacy of antiangiogenic therapies [29, 30]. Interestingly, we did not find a correlation of baseline levels or changes in levels of cytokines with aPTT. However, there was a significant association of increased PlGF levels at disease progression and prolonged aPTT (Figure 7F). In line with these observations, PlGF increased also in cediranib (pan-VEGFR-TKI) treated glioblastoma patients during therapy, and was associated with an improved clinical outcome [34]. Furthermore decreased levels of DKK3 at disease progression were associated with a prolonged aPTT. So far, DKK3 levels have not been assessed during antiangiogenic therapies. Recently, Guo et al. reported that DKK3 levels in RCC patients were significantly lower than those in healthy controls, which was suggested to reflect vessel normalization [35].

In addition, we observed that the levels of circulating cell populations like CEC and CEP significantly increased during therapy. However, the major CD45^−^CD31^+^ population remained relatively stable during evolvement of resistance, suggesting a balanced change of the subpopulations. This may contribute to evasive resistance or reflect the more chaotic tumor vasculature at disease progression [13]. In particular, CEP may generate new blood vessels in the tumor (vasculogenesis) whereas CEC could derive from shedded endothelial cells [13]. The change of CEC and CEP levels we observed in this study was comparable to other studies [13, 36, 37]. Moreover we found that other CEC subpopulations such as VEGFR2^+^CEC, whose pathophysiological role has not been investigated intensively so far, react differently to antiangiogenic therapies and decrease at the time point of resistance. Of note, we detected a strong baseline correlation between VEGF and CEP. This correlation underlines the role of VEGF as a key player in the recruitment of CEPs from the bone marrow [17]. Interestingly, at disease progression, although CEP and VEGF levels significantly increased, no association was detected. However, at disease progression CEP levels correlated with changes in PlGF levels (Table 2, only Pearson correlation significant, FDR value n.s.). In a previous report, PlGF was described as an important CEP mobilizing cytokine [17]. Hattori et al. showed that PlGF promotes recruitment of hematopoietic stem cells from a quiescent to a proliferative bone marrow microenvironment inducing differentiation and mobilization of bone marrow progenitor cells in mice [38]. Our data confirm these observations in a clinical setting and support the notion that CEP recruitment at disease progression is a multifactorial process, which might be independent from the presence of VEGF.

The potential of TEM expression levels in peripheral blood as markers of resistance during antiangiogenic therapy have not been assessed so far. The prognostic value of Robo4 expression has been analyzed in different tumors entities, however, with contradictory results. Recently, it has been shown that higher Robo4 expression levels in gliomas [39] and also acute myeloid leukemias [40] are associated with shorter overall survival. However, our group has previously found that higher TEM expression levels (Robo4, ECSCR, Clec14) correlated with a prolonged overall survival in primary lung cancer tumor samples [41]. Therefore, we hypothesized that higher TEM levels act as vascular stabilization factors and decrease the rate of metastatic spread. Here we report for the first time that levels of all three analyzed TEM behave similar during antiangiogenic therapy and decrease at disease progression (Figure 4). Furthermore, we could show that higher ECSCR and Robo4 expression at disease progression is associated with a prolonged aTTP (Figure 7C and 7D). These results suggest that loss of vascular stabilization factors could promote therapy resistance. In general, the concept of vascular stabilization by TEM is still controversial and currently no clear evidence in the clinical setting has been reported. However, further validation in a more homogenous and larger prospective patient cohort is warranted.

Furthermore, this study analyzed the changes of tumor blood perfusion by DCE-MRI. Although limited in sample size, the evaluable patient cohort (baseline *n* = 10) showed an increased perfusion/permeability during antiangiogenic therapy reflecting vessel normalization. At time of disease progression a significant change of contrast enhancement curves in tumor tissue from a type 3 to a type 1 curve was detected, indicating a reduction of tumor perfusion and permeability (Figure 6). Thereby the low permeability, which would be consistent with hypoxia [42], might reflect resistance to antiangiogenic agents. Furthermore, the concept of tumor vessel normalization, which theoretically induces a better tumor perfusion and nutrient supply, seems to be counterintuitive. However, tumor vessel normalization helps to improve the delivery of therapeutic drugs into tumor tissues, possibly leading to prolonged therapy responses by suppressing the pressure on tumor escape mechanisms [16, 43].

A major limitation of the presented study is the small sample size and the heterogeneous patient population included. However, the presented findings are clearly hypothesis generating. Nevertheless our findings provide a first indication of possible new biomarkers to monitor the effectiveness of antiangiogenic therapies. Furthermore, an extended biomarker profile panel including genetic analyses (like SNPs) or tumor tissue analyses would provide a deeper insight into resistance mechanisms.

## CONCLUSIONS

In summary, angiogenic cytokines were correlated with circulating cell populations and TEM expression levels, revealing that single marker analyses are insufficient for understanding the highly adaptive process of antiangiogenic resistance. We validated preclinically described resistance mechanisms in the clinical setting and showed for the first time that TEM might be new promising biomarkers. The observation that higher TEM expression is associated with prolonged aTTP could indicate more stabilized and functional vessels, which may have clinical and pathophysiological implications. Furthermore, sufficiently powered clinical trials are warranted to validate the findings of this study.

## PATIENTS AND METHODS

### Patients

Between November 2009 and July 2012 22 patients were included in this academic non-interventional pilot study. At the time of study closure 20 patients were evaluable for final analysis. Two patients had to be excluded because of missing follow-up visits (continuation of therapy in another hospital). All patients gave their informed consent and the study protocol was approved by the local institutional review-board (Ethics Committee of the Medical University of Innsbruck Number: UN3625_LEK, Eudract CT Number: 2008-008852-18, ClinicalTrials.gov Identifier: NCT01507740). Patients with HCC, RCC and NSCLC treated either with sorafenib, sunitinib or bevacizumab were eligible. An already ongoing antiangiogenic therapy was required for study inclusion (baseline analysis). Only patients with confirmed benefit (disease stabilization) from antiangiogenic could be included, while primary resistant patients were not part of the study. Laboratory examinations were part of the routine blood sampling and CT scans were performed as depended by clinical requirements. Functional imaging with dynamic contrast enhanced magnetic resonance imaging (DCE-MRI) was preplanned at study inclusion and at the time point of disease progression (Study plan shown in Figure 1).

Disease progression and response were recorded based on Response Evaluation Criteria in Solid Tumors (RECIST, version 1.0) and defined as stable disease (SD), partial response (PR) or progressive disease (PD). The time interval between start of the actual therapy and the first radiographic documentation of progression or death was defined as the clinical end-point (progression free survival [PFS]). As patients were prerequisite treated with antiangiogenic therapy at the time of study inclusion, we defined a modified endpoint entitled “time to progression during antiangiogenic therapy” (aTTP) describing the interval between baseline analysis (study inclusion) and the first documentation of progression or death. The primary objective of the trial was to delineate time to progression under antiangiogenic treatment (aTTP). Secondary endpoint was PFS.

### Peripheral blood mononuclear cells (PBMC) and serum analysis

To investigate the dynamic changes in circulating angiogenic factors, circulating endothelial cells (CECs), circulating endothelial progenitor cells (CEPs) and expression levels of TEM in peripheral blood we evaluated these profiles during therapy and at disease progression (every three to six months).

Serum samples were stored until investigation at −80°C. Customized protein arrays (Quantibody arrays, Ray Biotech) were utilized for analysis according to the manufacturer's protocol. All samples, quality controls, and standards were prepared as recommended with the supplied diluents and were processed in triplicate batches. Signals were assessed at 532 nm using Genepixx 4000B microarray scanner (Molecular Devices) and raw data were processed by using Q-Analyzer software provided by the company (Ray Biotech). Serum levels of vascular endothelial growth factor-A (VEGF), intracellular adhesion molecule1 (ICAM), platelet derived growth factor (PDGF), soluble vascular endothelial growth factor receptor2 (sVEGFR2), placental growth factor (PlGF), dickkopf3 (DKK3), monokine induced by gamma interferon (MIG), fibroblast growth factor (FGF), interleukine10 (IL-10), IL-12p40, IL-12p70, interferon gamma-induced protein 10 (IP-10) and hepatocyte growth factor (HGF) were measured.

### FACS analysis

Flow cytometric detection of CEC and CEP were carried out at the same day of blood sampling according to the protocol published by Duda *et al.* [44]. In brief, PBMC isolation was performed with FICOLL density gradient centrifugation and afterwards FC blocking was performed (Supplementary Figure 2). PBMC were incubated in triplicates with antibodies specific for CD31- FITC (BD Pharmigen), CD45- PerCP (BD Pharmigen), CD133- PE (MiltenyiBiotec), CD34- PC7 (Beckman Coulter), VEGFR2 (KDR)- PE (R & D Systems) and CD146- PE (BD Pharmigen). Appropriate fluorchrome-conjugated isotype-matched murine IgG antibodies (BD Pharmigen) were used as controls for each staining procedure. After incubation for 30 minutes at 4°C, cells were washed, resuspended and analyzed in a Cytomics-FC-500 cytometer using the Cytomics RXP-Software. CEC were defined as CD45^−^/CD31^+^/CD146^+^ cells and CEP were defined as CD45^−^/CD31^+^/CD133^+^ cells. Further we determined CD45^−^/CD31^+^/VEGFR2^+^ cells (cell gating and CEC phenotype definition depicted in Supplementary Figure 3).

### Quantitative real-time PCR (qPCR)

After PBMC isolation cells were preserved and stored in lysis buffer (Macherey Nagl RNA Isolation Kit, Düren, Germany). RNA was purified by using the Nucleo Spin RNA II kit (Macherey Nagel). Extracted total RNA was transcribed into cDNA using oligo-dT, hexanucleotide random primers and Super Script II Reverse transcriptase. PCR analysis was performed using 20 ng cDNA, Sybr-green Mix (Sensi Mix Plus SYBR Genxpress, Wien, Austria), and specific primers (10 pMol each). Analyses were carried out in a Corbett Rotor Gene 6.000 using Rotor Gene Software (Corbett Research, Cambridge, United Kingdom). The primers used for quantification were human Robo4 (5- GGATCACAGGAAGTGGAGGA; rev: 5-AACCCATTTGTTTGGCATGAG), Clec14 (5-AGAA GCTGGGAGAGACACCA, rev: 5-TGAGGAGTGGC AGAGGAAGT) and endothelial cell-specific chemotaxis regulator (ECSCR) (5-CAGCTGCCCTGTGACTACAA; rev: 5-CAGCAGCTGTCCATACAGGA). The TEM expression levels were normalized to the respective GAPDH expression levels (5-CATGACAACTTTGGTATCGTG, rev: 5- GTGTCGCTGTTGAAGTCAGA).

### Dynamic contrast enhanced magnetic resonance imaging (DCE-MRI)

DCE-MRI was performed using a 1.5 Telsa whole-body MR scanner (Magnetom Avanto, Siemens, Erlangen, Germany). DCE-MR images were obtained in axial orientation using a fast 3D T1 weighted (T1VIBE) gradient echo sequence with a temporal resolution of 6.2 seconds over a time period of about 10 minutes during uptake and wash-out of a gadolinium contrast agent (rate of injection: 0.1 mL/s Dotarem^®^). The parameters of the employed T1-weighted VIBE sequence were: repetition time (TR): 2.83 ms; echotime (TE): 1.02 ms; flip angle: 9°; slice thickness (SL): 4 mm; acquisition matrix: 256 × 96 interpolated to 256 × 192; field of view (FoV): 380 × 285 mm; number of slices: 24; acceleration factor (GRAPPA iPAT):2. Relative signal-change versus time curves were generated using ImageJ (Wayne Rasband, National Institutes of Health, Bethesda, MD, USA) by manual placement of elliptical regions of interest (ROI). The obtained enhancement curves were visually inspected for curve shape and maximum enhancement. Similar to methods used for DCE-MRI in prostate and breast cancer [45] the obtained curves were classified into three curve types: type 1 – progressive enhancement, type 2 – rapid enhancement with plateauing and type 3 - rapid enhancement followed by a rapid wash out of the contrast material [46]. Contrast enhancement curves are thought to be predictive for therapy resistance [47].

### Statistics

Using Wilcoxon test, we assessed which factors differed significantly between baseline and disease progression. The relationship between continuous variables was assessed by Pearson's correlation. False discovery rates (FDR) were calculated using the Benjamini and Hochberg method (presented as *q* values, online FDR calculator based on R code) [18]. PFS was analyzed by the Kaplan-Meier method and compared with the Log-rank test. To distinguish high vs. low levels of TEM expression levels a cut off was defined according the median qPCR TEM expression level. Statistical analyses of qPCR data were performed according to the delta Ct method described by Pfaffl [48]. Analyses were performed using GraphPad PrismTM6 (GraphPad Software Inc., La Jolla, CA). A *p*-value ≤ 0.05 was considered statistically significant. All values were presented as median ± range in the text and as mean ± standard error of the mean (S.E.M.) in the figures.

## SUPPLEMENTARY MATERIALS FIGURES


