# Supplementary material for: Biomarkers of evasive resistance predict disease progression in cancer patients treated with antiangiogenic therapies
Source: Oncotarget. 2016 Mar 4;7(15):20109–23. doi: 10.18632/oncotarget.7915 (PMC4991441; doi:10.18632/oncotarget.7915)
Supplement: Supplementary file 1 [file oncotarget-07-20109-s001.pdf]

Biomarkers of evasive resistance predict disease progression in cancer patients treated with antiangiogenic therapies

Supplementary Materials

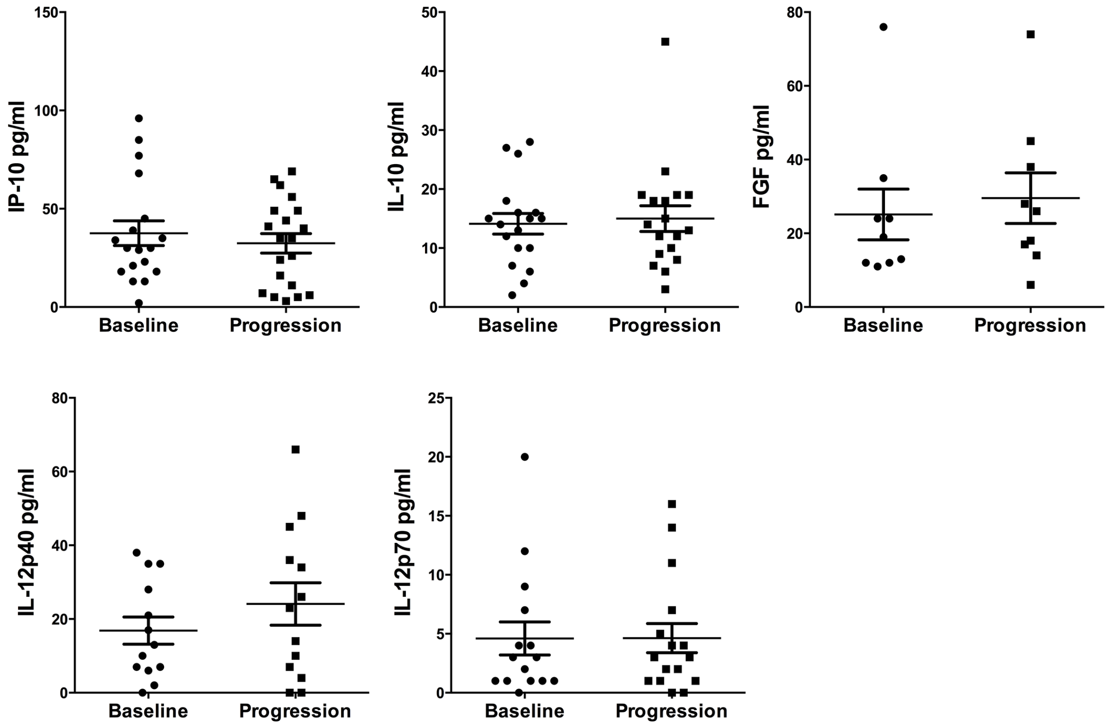

Supplementary Figure S1: Cytokine analyses comparing baseline investigations with disease progression. X-axis depicts the time points of measurements at baseline versus disease progression. Y-axis depicts the measured cytokine in picograms per milliliter (pg/ml). *Abbreviations:* Interleukine10 (IL-10), IL-12p40, IL-12p70, interferon gamma-induced protein 10 (IP-10) and fibroblast growth factor (FGF).

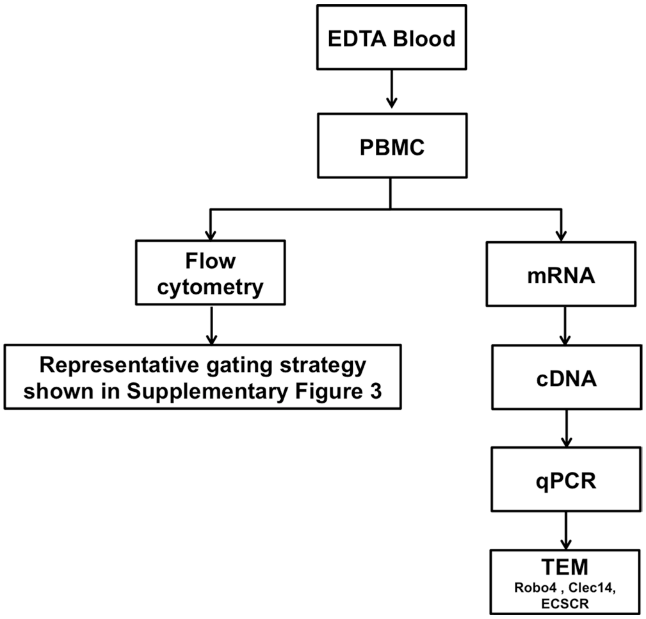

Supplementary Figure S2: Workflow for EDTA blood preparation for circulating endothelial cell populations and tumor endothelial marker (TEM) quantification.

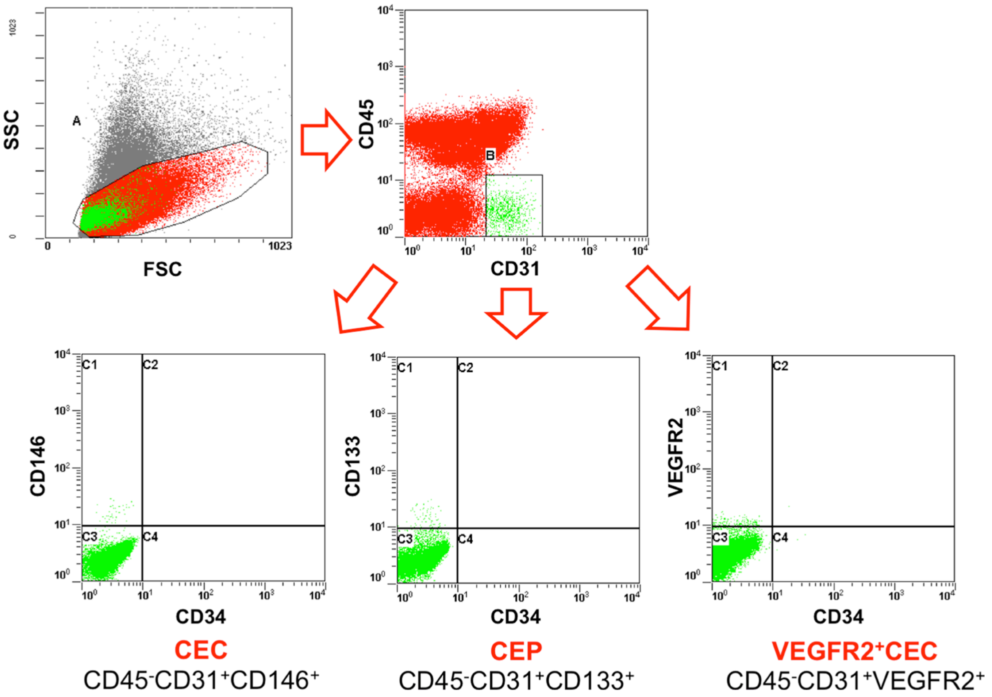

Supplementary Figure S3: Representative FACS gating strategy for CEP, CEC and VEGFR2$^+$CEC is shown. CD45$^-$ PBMCs were selected for CD31 expression followed by gating of defined subpopulations as CEC, CEP and VEGFR2$^+$CEC. *Abbreviations:* peripheral blood mononuclear cells (PBMC), circulating endothelial cells (CEC), circulating endothelial progenitor cells (CEP), vascular endothelial growth factor receptor2 (VEGFR2).
